# PM_2.5_-Bound Toxic Elements in an Urban City in East China: Concentrations, Sources, and Health Risks

**DOI:** 10.3390/ijerph16010164

**Published:** 2019-01-08

**Authors:** Lili Du, Yan Wang, Zhicheng Wu, Chenxiao Hou, Huiting Mao, Tao Li, Xiaoling Nie

**Affiliations:** 1 School of Environmental Science and Engineering, Shandong University, Qingdao 266237, China; lilydulili@126.com (L.D.); ZhichengWu1990@163.com (Z.W.); litao0201@gmail.com (T.L.); niexiaoling2015@126.com (X.N.); 2 Environmental Monitoring Central Station of Shandong Province, Jinan 250101, China; 18363518268@163.com; 3 Department of Chemistry, State University of New York College of Environmental Science and Forestry, Syracuse, NY 13210, USA; hmao@esf.edu

**Keywords:** elemental composition, PMF, human exposure, fine particles

## Abstract

Concentrations of PM_2.5_-bound trace elements have increased in China, with increasing anthropogenic emissions. In this study, long-term measurements of PM_2.5_-bound trace elements were conducted from January 2014 to January 2015 in the urban city of Jinan, east China. A positive matrix factorization model (PMF) and health risk assessment were used to evaluate the sources and health risks of these elements, respectively. Compared with most Chinese megacities, there were higher levels of arsenic, manganese, lead, chromium, and zinc in this city. Coal combustion, the smelting industry, vehicle emission, and soil dust were identified as the primary sources of all the measured elements. Heating activities during the heating period led to a factor of 1.3–2.8 higher concentrations for PM_2.5_ and all measured elements than those during the non-heating period. Cumulative non-carcinogenic and carcinogenic risks of the toxic elements exceeded the safety levels by 8–15 and 10–18 times, respectively. Arsenic was the critical element having the greatest health risk. Coal combustion caused the highest risk among the four sources. This work provides scientific data for making targeted policies to control air pollutants and protect human health.

## 1. Introduction

PM_2.5_ (particulate matter with an aerodynamic diameter < 2.5 μm) is a crucial air pollutant that is mainly emitted by human activities worldwide, especially in China [1]. Compared to coarse particles, the smaller size of PM_2.5_ allows for easier penetration into human lungs, which relates to long-term adverse human health effects such as cardiovascular disease and respiratory disease [2,3]. PM_2.5_ has been acknowledged as Group 1 carcinogens for human beings by the International Agency for Research on Cancer [4].

Although trace elements represent a small portion of PM_2.5_ mass, they play an important role in human health because of their high toxicity [5,6,7]. Trace elements can be accumulated in an organism via two primary exposure pathways (i.e., ingestion and inhalation); they can neither be digested nor biodegraded in most cases and are linked to ischemic heart disease, lung cancer, and chronic obstructive pulmonary disease after reaching a certain dose [8]. For instance, lead (Pb) can affect children’s nervous systems and impair brain development [9,10]; arsenic (As) exposure may cause various kinds of cancer, pigmentation disorders, and other related diseases [11]. Exposure to toxic elements in childhood can be connected with lower adult intelligence quotient scores and many diseases in, for example, cognitive function, which can alter the trajectory of one’s life [12,13,14].

Over the past decades, PM_2.5_ concentrations in China have been reported to be significantly higher than that in Europe and America [15,16]. Numerous studies have been carried out in China to understand the chemical composition, sources, and transport of PM_2.5_ [17,18,19]. Gu et al. reported an annual mean PM_2.5_ concentration of 59.31 μg/m^3^ in China in 2013, with the highest concentration in the North China Plain (101.6 μg/m^3^) [20]. PM_2.5_-bound toxic elements also typically have notably high concentrations in Chinese megacities and even rural areas. Concentrations of As, Mn, Cu, Zn, Cd, and Pb in Shanghai, Wuhan, and Beijing, and even background sites in China were 3–30 times that in New York and California [21,22,23,24,25]. In the last 30 years, the anthropogenic emissions of Cd, Cr, and Ni have increased threefold, and the pollution of Cr, As, and Cd has become rather serious [26]. These toxic elements are mainly contributed by coal combustion and traffic [27]. A large number of cities in China, especially in east China, are suffering serious PM_2.5_ pollution due to rapid economic development and high emission levels [28], and the abundant toxic elements therein are represent a severe threat to human health. Therefore, it is of great importance to conduct detailed studies on PM_2.5_ and its toxic effects in China in order to assess their health risks.

In this study, Jinan in east China was selected as an urban case to assess toxic elements in PM_2.5_. The annual average concentrations of PM_2.5_ was 168.85 μg/m³ in 2010 [29]. Although Jinan is not a megacity, environmental pollution has become a very important issue. Therefore, it is extremely urgent to assess the risk of PM_2.5_ pollution. This study aimed to (1) quantify the concentrations of elements in PM_2.5_, (2) identify their primary sources, and (3) assess the health risks of PM_2.5_–bound toxic elements to humans.

## 2. Methodology

### 2.1. Sampling

**Figure 1:**
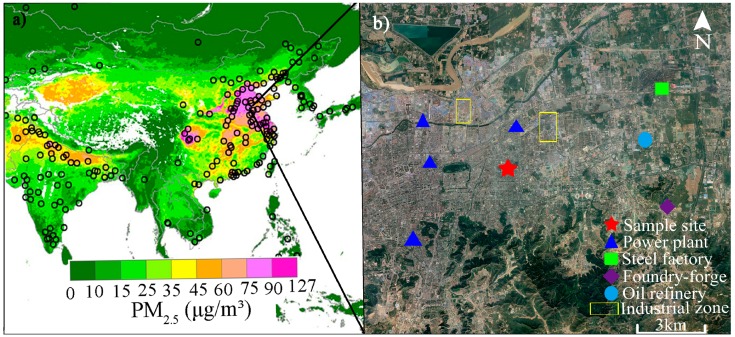
(**a**) Daily concentration of PM_2.5_ (µg/m^3^) in China [30], and (**b**) the locations of the sampling site (red star) and surrounding major anthropogenic emission sources (other shapes).

The sampling was conducted in Jinan, a typical industrial city in east China with heavy PM_2.5_ pollution (Figure 1). The sampling site was located on the rooftop of a four-story building (~15 m above ground level) in the campus of Shandong University (36°40′04″ N, 117°02′01″ E), 5 km away from the nearest power plant. The site is surrounded by densely populated residential areas and is impacted by nearby coal-fired power plants and industries.

A total of 176 PM_2.5_ samples were collected on 90-mm pre-heated quartz fiber filters (MUNKTELL, Sweden) using a medium volume particulate sampler (100 L/min). The sampling procedures were conducted every two days during non-rainy days from January 2014 to January 2015. The sampling time was from 8:00 a.m. to 7:30 a.m. on the next day. The entire sampling period (ESP) was divided into the heating period (HP, including January–March 2014 and November 2014–January 2015) and non-heating period (NHP, April–October 2014).

### 2.2. Laboratory Analysis

One-quarter of each sample filter or blank filter was digested in closed glass vessels with an acid mixture of 3:1 of 65% HNO_3_ and 37% HCl, using a hot plate at the temperature of 100 °C for 120 min in order to obtain the total concentrations of PM_2.5_-bound elements and reduce the loss of semi-volatile elements as much as possible. The inductively coupled plasma mass spectroscopy (ICP-MS; Agilent Technologies 7700, Santa Clara, CA 95051, USA) was used to analyze elements based on the the United States Environmental Protection Agency (US EPA) 200.8 method by using internal standard substances of Li, Sc, Ge, Y, In, Tb, and Bi to eliminate matrix interference. In total, 17 elements including Mg, Al, Ca, Cr, Mn, Fe, Co, Ni, V, Cu, Zn, As, Se, Sr, Cd, Ba, and Pb were measured. The detection limits varied from 0.05 µg/L for Cd to 7 µg/L for Fe, and the recoveries were within 73–93%.

### 2.3. PMF Source Apportionment

To classify the major sources of the elements in PM_2.5_, the US EPA positive matrix factorization (PMF) 5.0, an advanced multivariate receptor model, was used to estimate source profiles and contributions. PMF decomposed a matrix of sample data into two matrices: a factor contribution matrix and a factor profile matrix [31,32]. The principle can be expressed as follows:

(1) $$X_{ij}=\overset{p}{\underset{k=1}{\sum }}g_{ik}f_{kj}+e_{ij}$$

where *X_ij_* is the *j*th species concentration in the *i*th sample, *g_ik_* is the contribution of the *k*th factor to the *i*th sample, *f_kj_* is the *j*th species fraction from the *k*th source, *e_ij_* is the residual related to the *j*th species concentration measured in the *i*th sample, and *p* is the total number of independent sources. The object function *Q* is used to review the distribution of each species to evaluate the stability of the solution, which is defined as:

(2) $$Q=\overset{n}{\underset{i=1}{\sum }}\overset{m}{\underset{j=1}{\sum }}\left[\frac{X_{ij}-\sum_{k=1}^{p}g_{ik}f_{kj}}{uij}\right]$$

where *X_ij_* is the concentration of the *j*th species in the *i*th sample of the original data set, *p* is the number of factors, *g_ik_* is the contribution of *k*th factor to the *i*th sample, *f_kj_* is the fraction of the *k*th factor arising from species *j*, and *e_ij_* is the residual for each sample/species [33].

### 2.4. Health Risk Assessment

Although toxic elements constitute only a small proportion of the PM_2.5_ mass, they can have serious health impacts [2]. For an urban environment, the risks of toxic elements in PM_2.5_ to children and adults usually involve ingestion (i.e., ingesting PM_2.5_ through food, drinks, and anything that it is deposited on via hand-to-mouth activities) and inhalation exposure [34]. According to the exposure dose recommended by the US EPA, the chemical daily intake (CDI) through ingestion and the exposure concentration (EC) through inhalation were estimated to assess the exposure posed by toxic elements in PM_2.5_. According to the human health evaluation manual [34,35,36,37] and supplemental guidance for inhalation risk assessment [36], the values of CDI and EC were calculated as follows:

(3) $${\mathrm{CDI}}_{ing}=\frac{C_{m}\times \mathrm{EF}\times \mathrm{IngR}\times \mathrm{ED}\times \mathrm{CF}}{\mathrm{BW}\times \mathrm{AT}}$$

(4) $${\mathrm{EC}}_{inh}=\frac{C_{v}\times \mathrm{ET}\times \mathrm{EF}\times \mathrm{ED}}{\mathrm{ATn}}$$

where C_m_ is the arithmetic mean mass concentration of toxic elements in PM_2.5_ (mg/kg); C_v_ is the arithmetic mean volume concentration of toxic elements in PM_2.5_ (μg/m^3^); EF is the exposure frequency (180 days/year); IngR is the ingestion rate (200 mg/day for children and 100 mg/day for adults); ED is the exposure duration (6 years for children and 24 years for adults); CF is the conversion factor (10^−6^ kg/mg); BW is the average body weight (15 kg for children and 70 kg for adults); ET is exposure time (hours/day); AT is the averaging time (for non-carcinogens, AT = ED × 365 days; for carcinogens, AT = 70 year × 365 days); ATn is the average time (for non-carcinogens, ATn = ED × 365 days × 24 h/day; for carcinogens, ATn = 70 year × 365 days/year × 24 h).

The non-carcinogenic and carcinogenic risks of toxic elements in PM_2.5_ were quantified using the hazard quotient (HQ) and carcinogenic risks (CR), respectively. The non-carcinogenic risk was estimated using the HQ via ingestion (HQ_ing_) and HQ via inhalation (HQ_inh_). The carcinogenic risk was estimated using the CR via ingestion (CR_ing_) and CR via inhalation (CR_inh_). A hazard index (HI) is equal to the sum of multiple-chemical or multiple-route HQ. The equations were as follows:

(5) $${\mathrm{HQ}}_{ing}=\frac{{\mathrm{CDI}}_{ing}}{\mathrm{RfDo}}$$

(6) $${\mathrm{HQ}}_{inh}=\frac{{\mathrm{EC}}_{inh}}{{\mathrm{RfC}}_{i}\times 1000\mu g/\mathrm{mg}}$$

(7) $${\mathrm{CR}}_{ing}={\mathrm{CDI}}_{ing}\times \mathrm{SFo}$$

(8) $${\mathrm{CR}}_{inh}=\mathrm{IUR}\times {\mathrm{EC}}_{inh}$$

(9) $$\mathrm{HI}=\overset{n}{\underset{1}{\sum }}{\mathrm{HQ}}_{I}$$

where RfDo is the oral reference dose (mg/kg-day); RfC*_i_* is the inhalation reference concentrations (mg/m^3^); SFo is the oral slope factor ((mg/kg-day)^−1^); and IUR is the inhalation unit risk ((μg/m^3^)^−1^). The SFo, RfDo, RfC, and IUR were downloaded from the US EPA [38]. 

According to the risk management guidelines of the US EPA, the acceptable carcinogenic risk (as measured by CR) is between 1 × 10^−6^ and 1 × 10^−4^. For non-carcinogens, an HQ > 1 suggests the probability of adverse health effects, whereas an HQ < 1 indicates that the risk of non-carcinogenic effects is negligible.

## 3. Results and Discussion

### 3.1. PM_2.5_ and Element Concentrations

Daily PM_2.5_ concentrations during the ESP ranged from 1.6 to 653.5 μg/m³, with a mean value of 131.4 ± 84.5 μg/m³, slightly lower than that reported in 2010 (168.85 μg/m³) [29]. The annual average concentration of PM_2.5_ was 13 times the WHO air quality guideline (10 μg/m³). Only about a quarter of daily PM_2.5_ samples met the daily grade ΙΙ standard of Chinese ambient air quality—75 μg/m³ (GB 3095–2012). The PM_2.5_ concentrations were also higher than that at the adjacent background mountain site, Mt. Tai (40 μg/m³) [39], and that in Chinese megacities such as Beijing (102.4 μg/m³), Shanghai (52.3 μg/m³), and Guangzhou (44.2 μg/m³) [40,41,42], which suggested that there was much more severe PM_2.5_ pollution in this urban site. The concentrations of PM_2.5_ during the HP ranged from 14.9 to 656.5 μg/m³, with an average value of 163.2 μg/m³, evidently higher than that the NHP range of 1.6–260.1 μg/m³, with an average value of 95.7 μg/m³. Clearly, the PM_2.5_ pollution was much worse during the HP compared to the NHP, implying the more significant contributions of increased coal combustion for heating. 

**Figure 2:**
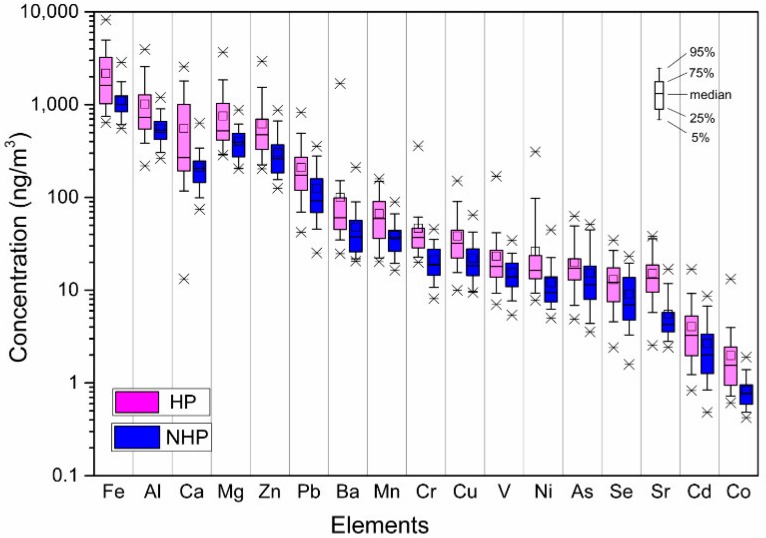
Concentrations of PM_2.5_-bound elements during the heating period (HP) and non-heating period (NHP).

Figure 2 shows the concentrations of 17 measured elements during the sampling period. Iron had the highest concentrations (1994 ng/m³ for the HP and 1067 ng/m³ for the NHP) and accounted for 38.3% and 35.5% of the total mass of the measured elements during the HP and NHP, respectively. The concentrations of Al, Mg, and Ca, which mainly originated from soils, accounted for 18.2%, 13.2%, and 9.7% of the total elements mass, respectively. The concentrations of other trace elements during the HP and NHP were both in an approximately descending order as follows: Zn > Pb > Ba > Mn > Cu > Cr > V > As > Ni > Sr > Se > Cd > Co. The order is similar with that at Mt. Tai (except Ni) [43]. However, the concentration levels of elements in Jinan (except Ca during the NHP) are elevated by 1.7–40 times compared to Mt. Tai. In addition, the concentrations of trace elements (e.g., Pb, Se, Zn, and Sr) and crustal elements (e.g., Ca, Mg, and Al) during the HP were generally higher than those during the NHP, probably due to increased coal combustion for heating and frequent dust storms in spring.

Compared to East Asian countries such as Japan and Korea, and urban cities in south China (e.g., Shenzhen and Hong Kong), the concentrations of PM_2.5_-bound elements (except V) in this site were significantly higher [44,45,46]. The concentrations of Mn, As, Pb, Cr, and Zn even exceeded those in Chinese megacities like Beijing, Shanghai, and Wuhan by a factor of 1.2–25 [23,47]. Intense industries and high coal consumption are supposed to be responsible for this severe pollution of trace elements. In comparison with the reported element concentrations during 2010 [29], we found Mg, Al, Ca, Mn, and Cu were reduced, whereas V, Cr, Co, and Ba were elevated in 2014, which was possibly related to changes of emission sources.

### 3.2. Source Apportionment

The PMF model was applied to identify the sources of 17 elements in PM_2.5_ during the ESP, HP, and NHP. The model was run with different numbers of factors using the random seed mode, and then the optimum solution with four factors was obtained. Each factor during the ESP, HP, and NHP had similar profiles of primary contributing elements (i.e., elements with high contribution percentages), indicating that the main sources of PM_2.5_-bound elements were constant during the ESP. Therefore, we will just discuss the source contribution profiles for the ESP below.

**Figure 3:**
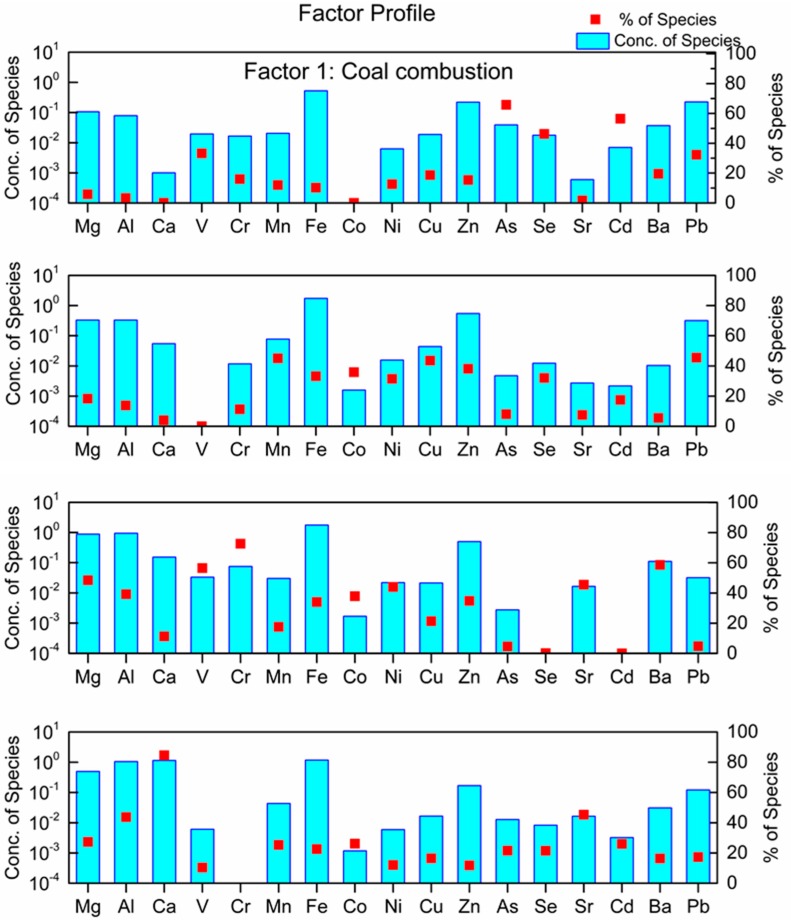
Four dominant factors contributing to PM_2.5_-bound elements using positive matrix factorization (PMF).

In Figure 3, Factor 1 is characterized by high loadings for Se, As, Cd, and Pb, indicative of coal combustion source [43,48]. In China, Se and As are typically emitted from coal combustion, a main source of these elements in atmospheric PM_2.5_ [49]. Most of the power supply for heating, electricity, and large-scale industry comes from coal combustion. Factor 2 is mainly defined by high loadings of Zn, Fe, Cu, and Mn, and moderate loadings of Pb, Ni, Se, and Co, which indicates smelting industry emissions [50]. Iron and steel smelting industries are significant contributors to Fe, Zn, Mn, Ni, and Cu [51], in agreement with the characteristics of an industrial city such as Jinan. Previous studies reported that industrial smelter was the source of Zn and Mn [52]. The main components defining Factor 3 are V, Cr, Ni, and Ba, which are closely associated with vehicle emissions [53]. Zereini et al. found high concentrations of Cr, Ni, and Zn in atmospheric particles at roadside, indicative of the dominant source of traffic [54]. Emissions from lubricating oil combustion by vehicles is an important source of atmospheric Ba [55]. In this study, the correlation (*r* = 0.83, *p* < 0.01) between Ni and V again suggested that they had the same source from heavy oil combustion [56,57]. Factor 4 is represented by high loadings of Ca, Mg, Al, and Sr, which are generally derived from soil dust [58,59]. As a developing city, there is constant pervasive construction activities every year, which is also a source of building dust. Overall, coal combustion, the smelting industry, vehicle emissions, and soil dust were identified to be major sources of measured elements in PM_2.5_, which accounted for 30.83%, 24.11%, 23.40%, and 21.66% of the PM_2.5_, respectively. The coal combustion in 2014 accounted for a smaller contribution compared with that in 2010 (38%), but it was still the dominated source. The contribution of vehicle emissions in 2014 was twice that in 2010 [29], probably induced by the rapid increase in the number of cars in the city and the accompanying rise in petrol consumption.

**Figure 4:**
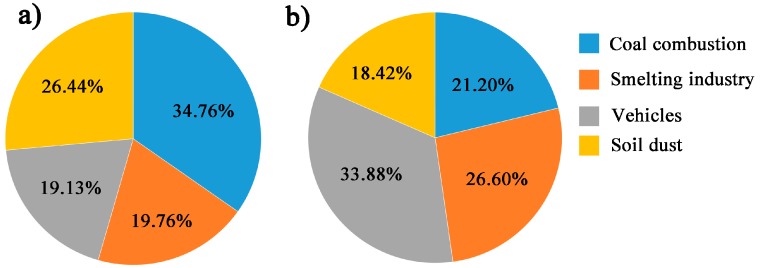
The contributions of the four sources to PM_2.5_-bound elements during the heating period (**a**) and non-heating period (**b**).

Figure 4 compares the contributions of the four sources to PM_2.5_-bound elements during the HP and NHP. Although the primary sources are the same, the relative contributions by individual sources between the HP and NHP are obviously different. During the HP, the contribution of coal combustion was 34.73% but vehicle emissions was 19.13%. The result was inverse during the NHP. This is a consequence of the increase in coal combustion during the HP and the relatively stable emissions from industries and vehicles during the whole year. Therefore, the contribution of coal combustion was reduced by 40% during the NHP, while the contribution from the smelting industry and vehicle emissions rose. A greater contribution by soil dust was found during the HP, possibly attributed to frequent dust storms in spring. In addition, the concentrations of PM_2.5_ and 17 elements contributed by each source during the HP were 1.3–10 times higher than during the NHP. This showed that although the percentage of contribution for vehicles and soil dust was reduced during the NHP, the contributing concentrations of them were higher than during the HP.

### 3.3. Health Risk Assessment

#### 3.3.1. Health Risks of Individual Toxic Elements

As previously mentioned, inhalation and ingestion exposure are typically the primary pathways for human exposure to airborne elements, which are assessed by non-carcinogenic and carcinogenic risk. Non-carcinogenic and carcinogenic risks values of PM_2.5_-bound toxic elements are listed in Table 1 and Table 2, respectively. 

The HQ values of Cr, Mn, Ni, Cu, Zn, As, Cd, and Pb in PM_2.5_ via ingestion and inhalation were quantified for adults and children (Table 1). The HQs via ingestion for both children and adults followed the variation pattern of As > Pb > Cr > Cu > Cd > Zn > Ni > Mn and the HQs via inhalation had the same trend (except Ni). The HQ_ing_ of As and Pb and the HQ_inh_ of As, Pb, and Mn were at unacceptable risk levels (>1) and the HQs of other toxic elements were acceptable. This also qualified the element As as being the most toxic element regarding non-carcinogenic risk, comprising 38–50% of the total risk to adults and children via ingestion and inhalation. Element Mn comprised 35% of the total risk via inhalation. Compared with adults, children had more risks from exposure. The HI for adults was 5.17 while it was 10.95 for children, both of which were higher than the established safe level but similar to a station near industrial activities in Spain [60]. It is evident that PM_2.5_-bound toxic elements in the study area posed an extremely large non-carcinogenic risk for residents, especially for children. The elements As, Pb, and Mn contributed to most of the non-carcinogenic value, so there is a need to prioritize their control. HI in the site was about twice that in Nanjing, primarily ascribed to the very high non-carcinogenic risks of As and Mn [61]. 

As shown in Table 2, the carcinogenic risk via ingestion was higher than inhalation. The CR_ing_ of Cr, Ni, and As were 2.0–8.5 times higher than the safe level (1 × 10^−4^) for adults and children, indicating that the three elements possibly have high risks for local residents. The CR_inh_ levels of Cr, Ni, As, Cd, and Pb were within the acceptable range. The total carcinogenic risks of five toxic elements for adults and children via ingestion were 7.95 × 10^−4^ and 17.5 × 10^−4^, respectively, far exceeding the acceptable risk range (1 × 10^−4^). The total risks via inhalation exposure were 1.18 × 10^−4^ and 2.96 × 10^−5^ for adults and children, respectively, presenting less risk than ingestion exposure. The total carcinogenic risks for adults and children via two exposure ways were 9.1 × 10^−4^ and 17.8 × 10^−4^, exceeding the maximum acceptable values of 8.1 and 16.8, respectively. This indicated that toxic element pollution in PM_2.5_ was extremely severe in the study area, as such, the cancer rate for local residents could increase with long-term exposure to the polluted air.

In short, the major toxic elements that caused health risks were As, Pb, Mn, and Ni, among which As was the most dangerous. People of different ages and occupations may spend different amounts of time outside. It was estimated that health risks increased by 12.5% for people who stayed outside for each 1 h more every day.

#### 3.3.2. Health Risks Associated with Each Sources

Table 3 sums the non-carcinogenic and carcinogenic risks from the four sources via ingestion and inhalation exposure to the selected element (Cr, Mn, Co, Ni, Cu, Zn, As, Cd, and Pb) in PM_2.5_. Detailed exposure assessments of the PM_2.5_ from specific sources may help to establish a better understanding of the exposure pathways as well as the detailed risk factors involved in both carcinogenic and non-carcinogenic risk. 

Among the four major sources, coal combustion contributed the highest non-carcinogenic via two exposure pathways (42%), because the risks of As and Pb were very high (in Section 3.3.1) and nearly 50% of As and Pb originated from coal combustion. The non-carcinogenic risks of other sources decreased in the order of the smelting industry > soil dust > vehicle emissions. The risk of the smelting industry was 1.9, accounting for 24% of the total contribution of the four sources. HQ_ing_ and HQ_inh_ of soil dust and vehicles were within the acceptable limit, therefore the potential health risk via the two pathways is almost negligible.

For carcinogenic risk, coal combustion and vehicles contributed 34% and 36% of the carcinogenic risk of the total source with the values of 3.64 × 10^−4^ and 3.91 × 10^−4^, respectively. The risk of the two sources were mainly from the exposure pathway of ingestion. Due to heavy traffic, Ni and Cr had high loadings for vehicles, as identified by PMF, contributing to 95% of the carcinogenic risk for vehicles. The value of the smelting industry risk was 1.84 × 10^−4^, to which Ni contributed 64%.

The above results showed that coal combustion emission was the most important source contributing to the high health risk, which means that the current energy structure is unconscionable. It is urgent to change the energy structure by using cleaner energy sources.

## 4. Conclusions

This study showed one-year measurements of PM_2.5_ and 17 elements in Jinan, an urban city in east China. The concentration of PM_2.5_ was 131.4 ± 84.5 μg/m³, with higher concentrations during the HP than NHP. Among the PM_2.5_-bound elements, Pb, Cr, and Zn exhibited extremely high concentration levels in comparison to Chinese megacities. Using PMF, four major sources were identified during ESP, including coal combustion (30.8%), industry (24.1%), vehicles (23.4%) and soil dust (21.6%). During NHP, the contribution of coal combustion was 40% lower compared to HP. 

The health risk assessment suggested that toxic elements in PM_2.5_ could pose serious non-carcinogenic and carcinogenic threats to residents, exceeding the safety levels by 4–16 times. Among the measured toxic elements, As posed the highest non-carcinogenic and carcinogenic risks. The non-carcinogenic risks for adults and children could result from the exposure to Pb, As, and Mn while carcinogenic risks came from Cr and Pb via ingestion. Coal combustion brought about the largest health risk among the four sources. With this in mind, coal combustion should be further controlled and reduced. 

Our group had analyzed the relationship between the concentration of air pollutants (PM_2.5_, SO_2_, and NO_2_) and hospital emergency room visits (ERVs) for respiratory diseases on the basis of a time series in the urban area of Jinan [62]. The increase in number of ERVs for respiratory illnesses was linked to the levels of air pollutants. PM_2.5_, which was positively and significantly associated with ERVs, had a more rapid impact and more significant effects on the number of ERVs than either SO_2_ and NO_2_. An increase of 1.4% (95% CI: 0.7%, 2.1%) in the number of ERVs for respiratory diseases was related to an increase of 10 µg/m^3^ in PM_2.5_ one day before. This work demonstrated the element As and coal combustion source to have the highest health risks. In the future, we plan to correlate the elemental composition of PM_2.5_ to ERVs for one or more respiratory diseases in an effort to evaluate the health risk assessment result and investigate which element(s) and emission source(s) do the most harm in order to provide scientific evidence for air pollution control. 

## Figures and Tables

**Table 1:** The values of hazard quotient via ingestion (HQ_ing_) and inhalation (HQ_inh_). HQ > 1 suggests the probability of adverse health effects.

Elements	HQ_ing_		HQ_inh_	
	Adults	Children	Adults	Children
Cr	0.393	0.831	0.359	0.540
Mn	0.015	0.315	1.10	1.65
Ni	0.062	0.131	0.406	0.610
Cu	0.234	0.496	ND	ND
Zn	0.063	0.132	ND	ND
As	2.59	5.49	1.19	1.79
Cd	0.166	0.352	0.069	0.100
Pb	1.65	3.49	ND	ND

ND: no data.

**Table 2:** The carcinogenic risks via ingestion (CR_ing_) and inhalation (CR_inh_). Acceptable values of CR are between 1 × 10^−6^ and 1 × 10^−4^.

Elements	CR_ing_		CR_inh_	
	Adults	Children	Adults	Children
Cr	1.94 × 10^−4^	4.27 × 10^−4^	8.62 × 10^−5^	2.15 × 10^−5^
Ni	3.79 × 10^−4^	8.37 × 10^−4^	3.34 × 10^−6^	8.36 × 10^−7^
As	3.83 × 10^−4^	8.46 × 10^−4^	2.64 × 10^−5^	6.60 × 10^−6^
Cd	ND	ND	2.14 × 10^−6^	5.34 × 10^−7^
Pb	ND	ND	7.17 × 10^−7^	1.79 × 10^−7^

ND: no data.

**Table 3:** The hazard quotient (HQ) and carcinogenic risks (CR) via ingestion and inhalation for Cr, Mn, Co, Ni, Cu, Zn, As, Cd, and Pb in PM_2.5_ associated with sources.

Sources	Non-Carcinogenic Risk		Carcinogenic Risk	
	HQ_ing_	HQ_inh_	CR_ing_	CR_inh_
Coal combustion	2.46	1.07	3.31 × 10^−4^	3.30 × 10^−5^
Smelting industry	1.18	0.77	1.72E × 10^−4^	1.36 × 10^−5^
Vehicles	0.58	0.69	3.25E × 10^−4^	6.54 × 10^−5^
Soil dust	0.95	0.60	1.28 × 10^−4^	6.78 × 10^−6^
